# Book Reviews

**Published:** 1894-12

**Authors:** 


### BOOK REVIEWS.

---

Medical Jurisprudence, Forensic Medicine, and Toxicology. By R. A. Witthaus, A.M., M.D., Professsor of Chemistry, Physics, and Hygiene in the University of the City of New York, etc., and Tracy C. Becker, A.B., LL.B., Counselor-at-law and Professor of Criminal Law and Medical Jurisprudence in the University of Buffalo. In four volumes. Volume II., illustrated with wood-cuts and three plates, two of them in colors. Sold by subscription only. William Wood & Co., 43 to 47 E. 10th Street, New York.

The first volume of this excellent work was reviewed in our June issue.

The present volume contains full and complete discussions of some of the most important medico-legal questions. The first eighty pages are devoted to the "Examination of Blood and other Stains," by Professor E. S. Wood, of Harvard University. The author describes the methods of making chemical, microscopic, and spectroscopic examinations of blood stains for purposes of diagnosis. The medico-legal relations of "Abortion and Infanticide" are ably discussed by Dr. J. C. Cameron, Professer of Obstetrics in McGill University. The signs and sequelæ of criminal abortion receive ample treatment. Under infanticide the main questions discussed are whether the child was born alive, and whether death was due to violence or natural causes. This section closes with concise directions for making an autopsy upon a new-born child for medico-legal purposes. "Pregnancy, Labor, and the Puerperal State" is the subject of one section, by Dr. J. C. Edgar, of New York. The average duration of pregnancy, diminished and protracted gestation, are questions of importance concerning the legitimacy of children; also, the period of early viability. These are all discussed in detail. The characteristics of the embryo and fetus during the several months of gestation, including the fully developed child, are given at some length. Other parts of this section discuss the signs of pregnancy (probable and certain), signs of recent delivery, superfetation, coffin birth, etc. Dr. Edgar and Dr. J. C. Johnston, of New York, contribute the part on "Rape." Under this head the signs of virginity are given in full, the greatest value being placed, of course, on the condition of the hymen. The authors incline to the negative in the oft-debated question, "Can a woman be violated against her will?" An interesting part of this division is a "Critical Study of Two Hundred Cases of Rape on Children in the City of New York" which presents some very instructive features. The part on "Railway Injuries," in their clinical and medico-legal relations, by Dr. W. B. Outten, of St. Louis, is almost a treatise on this one subject. Chapter I. of this division discusses the causes of railway accidents. Chapter II. describes the general nature of railway injuries; and chapter III. takes up specifically that one *bête noire* of the railway company, traumatic spinal neurosis. The long train of symptoms of this troublesome condition are taken up *seriatim*, and careful directions are given for examining persons supposed to be the subjects of "railway spine." There are also some considerations on the detection of malingering. Dr. Outten is a follower of Page, and takes a conservative view of railway back injuries; possibly too conservative. As to the nature of these injuries, he believes neither in the hysterical element of Charcot, nor in the neurasthenic of Oppenheim. The prognosis is uniformly favorable, he says, where "rest, isolation, and the proper surroundings" can be had; particularly if the claimant can make a satisfactory settlement with the company.

This review does not do justice to this volume, nor does it even allude to certain portions. We heartily recommend it to everybody. We have looked for too long to English authors for our works on Medical Jurisprudence. We think the editors and contributors to this treatise will make it an American reference-book of the highest authority.

Throughout the book numerous illustrative cases are reported under their proper headings.

A TEXT-BOOK OF MEDICAL CHEMISTRY, FOR MEDICAL AND PHARMACEUTICAL STUDENTS AND PRACTITIONERS. By Elias H. Bartley, B.S., M.D., Professor of Chemistry and Toxicology, and Lecturer on Diseases of Children, in Long Island College Hospital; Dean and Professor of Organic Chemistry, in the Brooklyn College of Pharmacy, etc. Third edition, revised and enlarged. Illustrated. Philadelphia: P. Blakiston, Son & Co., 1894.

The book before us, we note, has already passed through two editions, which would indicate that the work was one of considerable merit.

Following the introduction, which treats of the general properties of matter, and a short chapter on chemical physics, the author devotes forty-seven pages to the consideration of heat, light, electricity, and magnetism.

Next follows the consideration of the elements, which are grouped and considered in detail, according to Mendelejeff's table. This part of the book is well written, and there is little to which exception could be taken.

The portion of the work devoted to organic chemistry is also well written, and the classification and order, in which are considered the various complex substances which fall into the division of chemistry, is natural, and consequently presents the fewest possible obstacles to the beginner. The remainder of the book is taken up by physiological and clinical chemistry, the greater part of which deserves the highest praise, embodying, as it does, the latest and best.

The remaining chapters are devoted to the consideration of the urine and its abnormalities; while, as a whole, this is unusually comprehensive and clearly written, there are certain inaccuracies.

For instance, we challenge, as misleading, the statement made on p. 583 that, "as a rule, the urine of Bright's disease is of low specific gravity." A student, on reading the above, would erroneously infer that only in exceptional instances the urine of Bright's disease would be of a high specific gravity.

In speaking of the tests for biliary pigments in the urine, we note that the one devised by Huppert is not given; in our hands this list has given far better results than either of those recommended by the author.

We are pleased to see that the acetic acid and ferrocyanide of potassium test for albumen is commended, and, also, the phenylhydrazine and indigo-carmine test for glucose.

There is an appendix in which rules are given for the spelling and pronunciation of chemical terms, a glossary of unusual chemical terms, and a number of useful tables of weights and specific gravities and solubilities of various chemicals. The practice of printing the names of chemicals and definitions in heavy type adds to the usefulness of the book.

The publisher's work is well done. The book, on the whole, is a good one of its kind, and may be properly recommended to both students and practitioners of medicine and pharmacy. H. F. H.

---

PRACTICAL URANALYSIS AND URINARY DIAGNOSIS: A MANUAL FOR THE USE OF PHYSICIANS, SURGEONS, AND STUDENTS. By Charles W. Purdy, M.D., Professor of Urology and Urinary Diagnosis at the Chicago Post-Graduate Medical School. Author of "Bright's Disease and Allied Affections of the Kidneys"; also of "Diabetes: Its causes, Symptoms, and Treatment." With Numerous Illustrations, including Photo-Engravings and Colored Plates. Extra Cloth, $2.50 net. Philadelphia: F. A. Davis Co., Publishers, 1914 and 1916 Cherry Street.

This is the fullest and most comprehensive work on this subject that we know of. No one has given more time and study to uranalysis and urinary diagnosis than Dr. Purdy, and no one is better qualified to write a treatise such as this. The author has endeavored to bring out the chemical composition of the urine in its relation with physiological processes and pathological facts. In dealing with both normal and abnormal urine every constituent has been considered, with their chemical nature and composition, their sources in the economy, the significance of its presence, increase or decrease in the urine, and the most improved methods of their detection. All the tests for albumin and sugar and other abnormal constituents are carefully described, and the author's preferences stated.

The second division of the work—Urinary Diagnosis, aims at a description of those urinary features which indicate the presence of certain pathological conditions. Thus the clinical aspects of the urine in acute and chronic Bright's disease, diabetes (insipidus and mellitus), renal calculus, uremia, cystitis, tumors of the bladder, etc.; also the characters of urine in the infectious diseases, typhoid fever, diphtheria, scarlet fever, etc.; also the urine in diseases of the liver, nervous system, respiratory organs, etc. An excellent Appendix on the Examination of Urine for Life Insurance closes the volume, certainly one of the most valuable portions of the whole work. No medical library is complete without a work of this sort. There is none better than this.

---

A MANUAL OF THE PRACTICE OF MEDICINE, PREPARED ESPECIALLY FOR STUDENTS. By A. A. Stephens, A.M., M.D., Lecturer on Terminology and Instructor in Physical Diagnosis in the University of Pennsylvania; Physician to St. Mary's Hospital, to the Out-Patient Department of St. Agnes's Hospital, and to the Southeastern Dispensary, Philadelphia. Third Edition, revised, illustrated. W. B. Saunders, 925 Walnut Street, Philadelphia. Price, $2.50.

The fact that three editions of this book have been called for in two years is of itself a guarantee that it is one of the best manuals on the market. It is a neat, well bound book of five hundred pages, this not being too large for convenient use. It contains concise articles on all the most important diseases likely to be encountered by the general practitioner. These articles are short and to the point. The section on diseases of the skin is very good, and just the thing that students and general practitioners want. In preparing this book the author has consulted most of the late standard works on the practice of medicine. It is thoroughly up to date.

J. L. C.

---

A SYNOPSIS OF THE PRACTICE OF MEDICINE. By Wm. Blair Stewart, A.M., M.D., Lecturer on Therapeutics; Late Instructor in the Practice of Medicine in the Medico-Chirurgical College of Philadelphia; Demonstrator in the Philadelphia School of Anatomy, etc. E. B. Treat, 5 Cooper-Union, New York.

We have just received the above book from the publishers. It is a neat little book, well written, and well indexed. The introduction is well worth the price of the book, as it is very clear and concise in the definition of the Etiology, Pathology, and Symptoms of Disease, besides a great many other subjects relating to general medicine. The special pathology of the diseases described is good and full enough for a book of this kind, as it is not intended to take the place of the larger text-books, but for a reference book for busy practitioners. J. L. C.

---

Chemistry: General, Medical, and Pharmaceutical. By John Attfield, F.R.S., Professor of Chemistry to the Pharmaceutical Society of Great Britain, Editor of the British Pharmacopœia, etc. Fourteenth edition. Lea Bros. & Co., 706, 708 Sansom Street, Philadelphia.

Attfield's Chemistry is so well known, and in such general use, that no long review is necessary here. This (the fourteenth) edition is uniform with preceding issues, except that it is adapted to the United States pharmacopœia, which is a distinct advantage to us over here. Other improvements have also been made, in accordance with the advances in chemical and pharmacal science. For a practical manual on General, Medical, and Pharmaceutical Chemistry, we know of nothing superior to Attfield.

---

### A Famous Show of Beauty.

The show of distinguished beauty, transfixed by famous artists, which is now taking place at the Academy of Fine Arts in New York, has been anticipated by the *Cosmopolitan Magazine* in its November issue, in an article by Wm. A. Coffin, with illustrations of some of the more beautiful faces. The "Great Passion of History" series has for this month's subject the romantic career of Agnes Sorel, who influenced the destinies of France under Charles VII. "The Art Schools of America," "The Great British Northwest Territory," "The Chiefs of the American Press," and the "Public Library Movement," are amongst the *Cosmopolitan's* table of contents. Survivors of the war and their children will find intense interest in "The Story of a Thousand," a personal narrative begun in this number by Albion W. Tourgee, who tells in a graphic way, of a regiment which saw fierce service—of its organization, its marches, its sports, and its death-roll.

